# Preparations of Polyurethane Foam Composite (PUFC) Pads Containing Micro-/Nano-Crystalline Cellulose (MCC/NCC) toward the Chemical Mechanical Polishing Process

**DOI:** 10.3390/polym16192738

**Published:** 2024-09-27

**Authors:** Yi-Shen Huang, Yu-Wen Huang, Qiao-Wen Luo, Chao-Hsing Lin, Penjit Srinophakun, Supanicha Alapol, Kun-Yi Andrew Lin, Chih-Feng Huang

**Affiliations:** 1 Department of Chemical Engineering, i-Center for Advanced Science and Technology (iCAST), National Chung Hsing University, Taichung 40227, Taiwan; yishen617@gmail.com (Y.-S.H.); s26005916@gmail.com (Q.-W.L.); 2 Semiconductor and Green Technology Program, Academy of Circular Economy, National Chung Hsing University, Nantou City 540216, Taiwan; oooo321280@gmail.com; 3 IV Technologies Co., Ltd., Taichung 40755, Taiwan; eddie.lin@ivt.com.tw; 4 Department of Chemical Engineering, Faculty of Engineering, Kasetsart University, Bangkok 10900, Thailand; fengpjs@ku.ac.th (P.S.); supanicha.a@ku.th (S.A.); 5 Department of Environmental Engineering, Innovation and Development Center of Sustainable Agriculture & Research Center of Sustainable Energy and Nanotechnology, iCAST, National Chung Hsing University, Taichung 40227, Taiwan

**Keywords:** polyurethane foam, microcrystalline cellulose, nanocrystalline cellulose, polishing pads

## Abstract

Polyurethane foam (PUF) pads are widely used in semiconductor manufacturing, particularly for chemical mechanical polishing (CMP). This study prepares PUF composites with microcrystalline cellulose (MCC) and nanocrystalline cellulose (NCC) to improve CMP performance. MCC and NCC were characterized using scanning electron microscopy (SEM) and X-ray diffraction (XRD), showing average diameters of 129.7 ± 30.9 nm for MCC and 22.2 ± 6.7 nm for NCC, both with high crystallinity (ca. 89%). Prior to preparing composites, the study on the influence of the postbaked step on the PUF was monitored through Fourier-transform infrared spectroscopy (FTIR). After that, PUF was incorporated with MCC/NCC to afford two catalogs of polyurethane foam composites (i.e., PUFC-M and PUFC-N). These PUFCs were examined for their thermal and surface properties using a differential scanning calorimeter (DSC), thermogravimetric analysis (TGA), dynamic mechanical analyzer (DMA), and water contact angle (WCA) measurements. *T*_g_s showed only slight changes but a notable increase in the 10% weight loss temperature (*T*_d10%_) for PUFCs, rising from 277 °C for PUF to about 298 °C for PUFCs. The value of Tan δ dropped by up to 11%, indicating improved elasticity. Afterward, tensile and abrasion tests were conducted, and we acquired significant enhancements in the abrasion performance (e.g., from 1.04 mm/h for the PUF to 0.76 mm/h for a PUFC-N) of the PUFCs. Eventually, we prepared high-performance PUFCs and demonstrated their capability toward the practical CMP process.

## 1. Introduction

Polyurethane (PU) is one of the most versatile polymeric materials that could be produced as thermoplastic polyurethane (TPU), polyurethane foam (PUF), and fiber-reinforced PU composites featuring applications such as aerospace, automotive, coatings, textiles, elastomers, and encapsulation of electronics [1,2,3,4,5]. TPU is a linear copolymer with alternating flexible and rigid segments, offering adjustable hardness and elasticity. The flexible segments, derived from polyether or polyester polyols like polyethylene glycol [6,7], enhance elasticity, while the rigid segments, formed by urethane groups, increase hardness and strength through intermolecular hydrogen bonding [8]. This structure gives TPU its unique thermomechanical properties, wear resistance, chemical resistance, and ease of processing [9]. In the second case, thermosetting polyurethane, with its 3D foam structure (i.e., PUF), maintains good storage modulus and stability at high temperatures, making it ideal for PUF applications. Among the applications, the chemical mechanical polishing (CMP) process is particularly significant, as it is used to clean and planarize materials like dielectrics, semiconductors, and metals [10,11,12,13]. CMP is crucial in the semiconductor industry for creating multilayer nanometer-width integrated circuits by precisely controlling layer thickness on wafers. In the CMP process, precise thickness removal depends on key components like the polishing head, diamond tip, polishing pad, and slurry. The polishing pad, typically made of PUF, effectively delivers the slurry. PUF is favored for its compressibility, hardness, corrosion resistance, and uniform porous structures [14,15,16]. The environmental impact of CMP is under scrutiny due to increased wafer demand, as consumables like slurries and pads are non-reusable.

PUF’s durability enhances the lifespan of polishing pads and fiber-reinforced PUF composites with their superior abrasion resistance. One representative example is the incorporation of nanocellulose fiber interactions, showing promise for durable pads [17,18]. Additionally, incorporating biomass into PUF, such as nanocellulose, offers a sustainable alternative, combining high durability with environmental benefits. Among nanocellulose fibers, microcrystalline cellulose (MCC), and nanocrystalline cellulose (NCC) are well known for their high crystallinity, which contributes to their strength and stiffness, enhancing the mechanical properties of polymer matrix [19,20,21,22,23,24]. Due to their dimensions, MCC and NCC have large surface areas, providing a high aspect ratio that improves the barrier properties of polyurethane coatings against corrosion. The hydrophilicity of MCC and NCC can be challenging to incorporate into non-polar polymer matrices, but their dispersion methods allow for effective reinforcement in polymer matrices. Martin et al. reported that nanocellulose-toughened TPU composites improved tensile strength by up to 43% without compromising elastic properties. They utilized a simple method to prepare well-dispersed nanocellulose/polyol solutions for a scalable reactive extrusion process to afford TPUs. The addition of nanocellulose enhances phase mixing in TPU, raising the primary relaxation temperature due to hydrogen bonding [25]. Kalappa et al. synthesized nanocellulose-based polyurethane foams, which showed improved thermal stability, with initial degradation temperature increasing from approximately 256 to 271 °C. However, tedious treatments on the nanocelluloses were applied [26]. Aboelenien et al. studied PU/NCC composites for coatings, finding that they increased corrosion resistance [27]. Therefore, the PUF and MCC/NCC preparations for applying the CMP process are still limited.

**Scheme 1:**
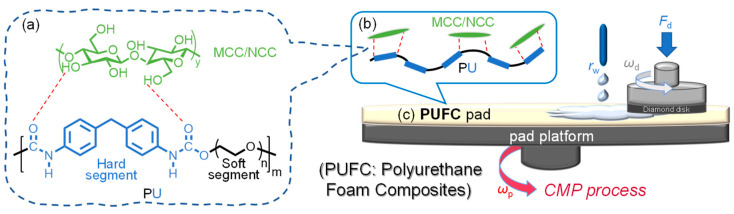
Preparations of PUFC pad for the applications in the simulated CMP process (PUFC: polyurethane foam composite; MCC/NCC: micro-/nano-crystalline cellulose; *F*_d_: downforce on the diamond disk; *ω*_d_: rotating speed of the diamond disk; *r*_w_: water flow rate; *ω*_p_: rotating speed of PUFC pads).

Our novel solution is to use a simple method of preparing well-dispersed MCC/polyol and NCC/polyol solutions and avoid tedious treatments on the MCC and NCC. As shown in Scheme 1, we employed MCC and NCC as fillers to synthesize two PUF composites (i.e., PUFC-M and PUFC-N series) for polishing pads. Firstly, we characterized the MCC and NCC through SEM and XRD. Then, we synthesized the pristine PUF and PUFCs using the mechanical stirrer under ambient conditions. The complete reaction was confirmed by FTIR analysis. We further characterized these specimens’ thermal and mechanical properties through TGA, DSC, DMA, and tensile tests. The hydrophobicity of the specimen surface was qualified through water contact angle measurements. Eventually, the abrasion resistance of the pristine PUF and PUFCs was evaluated by imitating the polishing tests.

## 2. Materials and Methods

### 2.1. Symbol Details

Abbreviations and symbols are listed as follows: microcrystalline cellulose (MCC), nanocrystalline cellulose (NCC), ethylene glycol (EG), methanol (MeOH), tetrahydrofuran (THF), polyurethane foam (PUF), PUF composite (PUFC), relative conversion (rConv), storage modulus (E′), water contact angle (WCA), maximum tensile stress (σ_m_), maximum tensile strain (ε_m_), toughness (*U*), instantaneous abrasion rates (*r*_inst. abra._), overall abrasion rates (*r*_overall abra._), abrasion depth (*D*_abra._), and abrasion time (*t*_abra._).

### 2.2. Materials

IV Technologies Co., Ltd. (Taichung City, Taiwan) provided C0-IS02024 (isocyanate, 99.5%, viscosity = 180 cPs) and C0-PO2024 (polyol, 99.5%, viscosity = 4300 cPs). Industrial Technology Research Institute (ITRI, Hsinchu City, Taiwan) provided MCC (99%) and NCC (99%). Acetone (99.9%), EG (99%), MeOH (99.9%), and THF (99%) were purchased from Sigma-Aldrich (St. Louis, MI, USA), and all materials were used without purification.

### 2.3. Preparing PUF and PUF Incorporated with MCC/NCC

The pristine PUF, PUF/MCC, and PUF/NCC composites were synthesized using the commercial product of polyol and isocyanate in a weight ratio of 20 g:10 g. In the case of pristine PUF, the isocyanate was preheated at approximately 35 °C and blended with polyol. A disk-shaped mold with an interior dimension of diameter of 30 cm and thickness of 6 mm was coated with a thin layer of releasing agent and pre-heated at approximately 60 °C. The PU monomer blends were poured into the mold and sealed for foaming in a few minutes. The de-molded PUF was then postbaked in a 60 °C oven for a desired period.

For the preparations of PUF/MCC and PUF/NCC composites (i.e., named as PUFC in general), vacuum-dried MCC/NCC loading 0.1, 0.3, and 0.5 phr was added into the polyol. The mixture was stirred using a high-speed stirrer (ca. 3000 rpm) for a few minutes. Isocyanate was preheated in a reactor at approximately 35 °C and then blended with polyol/MCC or polyol/NCC) mixtures using the stirrer above. The mixture blends were quickly poured into the pre-heated disk mold with a release agent coating and foaming for a desired period at approximately 60 °C. The de-molded PUFC specimens were postbaked in a 60 °C oven for another desired period. The prepared PUFC were separately named PUFC-M# and PUFC-N#, where # represents the added amounts (# = 0.1, 0.3, and 0.5 (phr)). Table S1 (see the Electronic Supporting Information (ESI)) summarizes all the samples for investigations.

### 2.4. Characterization

SEM images were acquired using a JEOL JSM 7401F FE-scanning electron microscope operated at an accelerating voltage of 100 kV. X-ray diffraction (XRD) spectra were recorded over diffraction angles (2θ) from 10 to 40° using a Bruker AXS D8 Discover SSS high-resolution X-ray diffractometer and Ni-filtered Cu Kα radiation (λ = 0.1548 nm) at 40 kV and 40 mA. Fourier-transform infrared spectroscopy (FTIR) spectra were recorded using a PerkinElmer Spectrum One FT-IR spectrometer equipped with an attenuated total refraction stage and operated at a resolution of 4 cm^−1^ with 24 scans. Accordingly, the relative conversion (rConv) can be estimated from the representative intensities of the isocyanate peak from the MDI monomer (ca. 2270 cm^−1^) and the carbonyl peak from the PU backbone (ca. 1710 cm^−1^) by the following equation: rConv = (*I*_1710_)/(*I*_1710_ + *I*_2270_). Using a differential scanning calorimeter (DSC, TA Instruments DSC 2010), the glass transition temperatures (*T*_g,DSC_) of the pristine PUF and PUFCs were obtained under N_2(g)_ (first run to 160 °C then 5 min isothermal; second run from 0 to 180 °C with a ramping of 10 °C/min). The thermal stability of the pristine PUF and PUFCs was obtained by thermogravimetric analysis (TGA) using a TA Instruments Q50 analyzer (from 50 to 700 °C under N_2_(g) with a ramping of 20 °C/min). The storage modulus and *T*_g,DMA_ of the pristine PUF and PUFCs were conducted using a dynamic mechanical analyzer (DMA) using a Perkin Elmer DMS6100 analyzer (from 25 to 150 °C under N_2(g)_ with a ramping of 5 °C/min at 1 Hz). A GOTECH AI-3000 tensile test machine recorded the tensile stress–strain curves of specimens with a cell with a maximum load of 500 kg. Dumbbell-shaped specimens of the pristine PUF and PUFCs with dimensions of 20 mm, 5 mm, and 3 mm (gauge length, width, and thickness) were fixed between the clamps. The tensile speed was controlled at 500 mm/min during the tests. Water contact angles (WCAs) were acquired using a First Ten Ångstrom FTA-1000B analyzer. Dried samples were pressed at ca. 50 psi to obtain flat surfaces under ambient and dried in a vacuum prior to measuring WCAs. Abrasion tests of the pristine PUF and PUFCs were conducted by a Presi Mecatech 334 SPC polishing machine. The specimens were fixed on the lower polishing platform. As shown in Scheme 1, a press force of 7 daN (*F*_d_) and a rotating speed of 150 rpm (*ω*_d_) were applied to the upper diamond disk. The polishing test was conducted using a 30 cm polishing pad with a rotating speed of 250 rpm (*ω*_p_) under DI water rinsing flow (*r*_w_ = 0.6 L/min, 50 °C). The wear rate was estimated by monitoring the residual thickness of specimens per 0.5 h using the following equation:

$$\mathrm{Abrasion}\;\mathrm{rate}\;(r_{\mathrm{abra}.})=\frac{\mathrm{Abraded}\;\mathrm{thickness}\;\left(\mathrm{mm}\right)}{\mathrm{Abrasion}\;\mathrm{time}\;\left(h\right)}$$

### 2.5. Research Workflow

**Chart 1:**
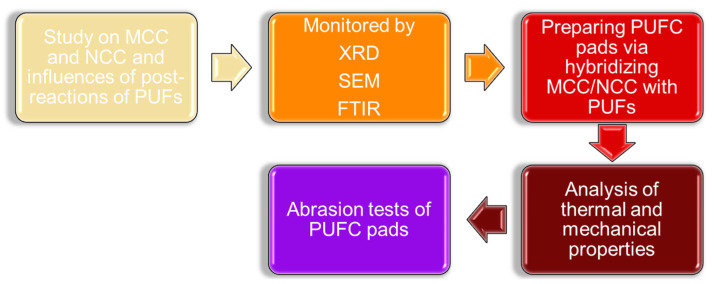
Our overall experimental design and workflow.

Chart 1 represents our overall experimental design and workflow in this study.

## 3. Results and Discussion

### 3.1. Characterization of MCC and NCC and Influence of Postbaked on PU Pads

**Figure 1:**
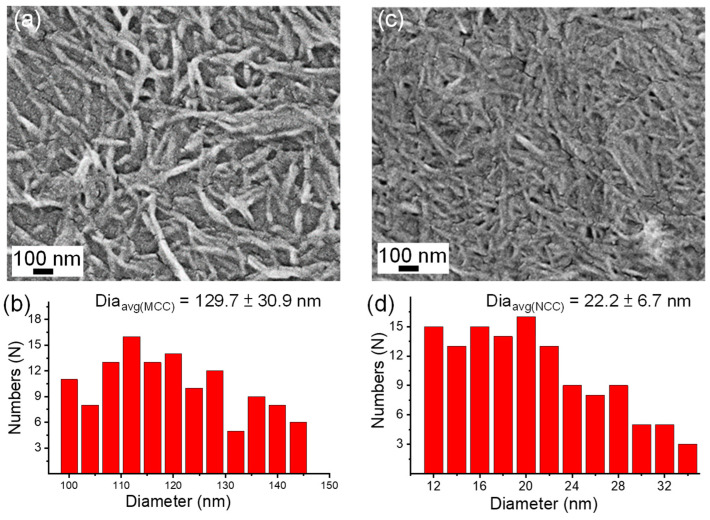
SEM images (×50,000) and statistics analysis (N_total_ = 125) of (**a**,**b**) MCC and (**c**,**d**) NCC.

Figure 1 shows the SEM images for the characterization of MCC and NCC, which depict the structures of MCC and NCC and reflect their different preparations. In Figure 1a, the fibers have an average diameter of 129.7 ± 30.9 nm, displaying significant variability in width, as evidenced by the histogram below (i.e., Figure 1b), which shows a wide distribution of fiber widths. Conversely, Figure 1c reveals nanocellulose crystals with a much smaller average diameter of 22.2 ± 6.7 nm, exhibiting a more uniform structure with less variability in fiber width, as shown by a narrower distribution in the corresponding histogram (i.e., Figure 1d). These images illustrate that NCC has a more similar and reduced fiber size (approximately 6 times less) than MCC.

**Figure 2:**
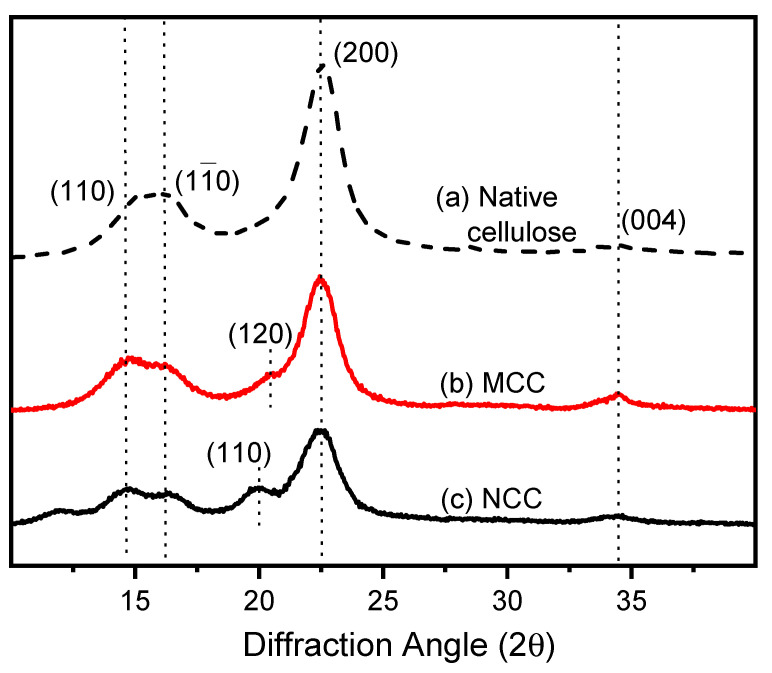
XRD patterns with a 2θ range of 10–40° for (**a**) native cellulose, (**b**) MCC, and (**c**) NCC powders.

Then, the MCC and NCC were examined by XRD. As shown in Figure 2, the XRD pattern exhibits the typical peaks associated with cellulose I structures, specifically cellulose I_β_. The peak at 14.9° corresponds to the (1 0 0) plane of cellulose I_α_ or the (1 −1 0) plane of cellulose I_β_, indicating the presence of these polymorphs in the sample. The peak at 16.7° is associated with the (0 1 0) plane of cellulose I_α_ or the (1 1 0) plane of cellulose I_β_, suggesting the predominant crystalline structure is cellulose I_β_. We observed a shoulder peak in the MCC and NCC at 20.4° and 20.0° for the (1 2 0) and (1 1 0) planes, respectively [28,29,30]. The prominent peak at approximately 22.6°, corresponding to the (2 0 0) plane of cellulose I_β_, signifies a high crystallinity index [31,32]. The crystallinity index (Cr.I. (%)) and the crystal unit sizes (Cr.U (nm)) can be further estimated [33,34,35]. From pattern a, the Cr.I. and Cr.U. of native cellulose are 81.3% and 4.5 nm. From pattern b, the Cr.I. and Cr.U. of the MCC are 92.3% and 5.3 nm. From pattern c, the Cr.I. and Cr.U. of NCC are 85.7% and 4.7 nm. These results suggest that the MCC and NCC possess high crystallinity, with the primary crystal structure being cellulose I_β_. The XRD pattern highlights the sample’s crystalline nature, showing well-defined peaks corresponding to the characteristic planes of cellulose I structures.

**Figure 3:**
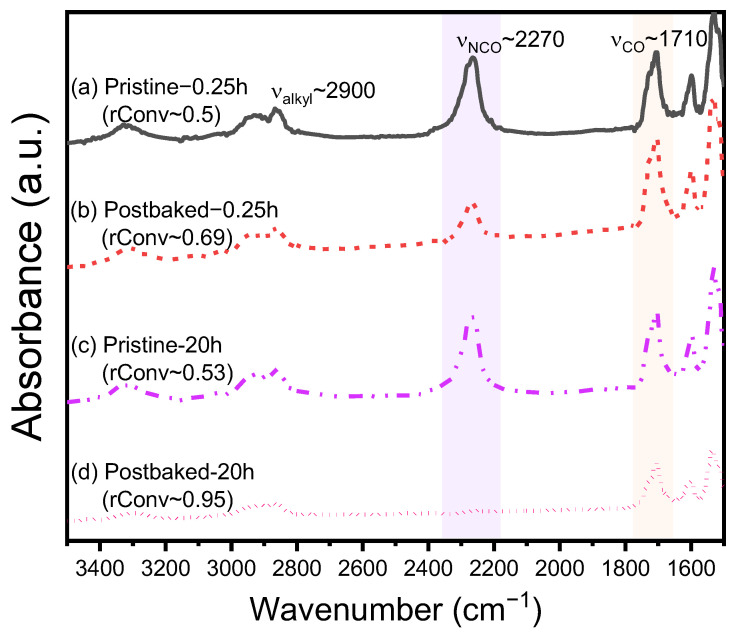
FTIR spectra (3500–1500 cm^−1^) of as-synthesized (i.e., (**a**,**c**) Pristine) and after postbaked (i.e., (**b**,**d**) Postbaked) for PUF samples in various treating times [rConv = (*I*_1710_)/(*I*_1710_ + *I*_2270_)].

Before preparing the composites of PUF and MCC/NCC, various polymerization and postbaked treatment times on PUF at 60 °C were investigated by FTIR to comprehend the processes that influence the changes in the chemical structure. Figure 3 shows FTIR spectra of four samples: (a) Pristine-0.25 h (i.e., synthesis of PUF in 0.25 h), (b) Postbaked-0.25 h (i.e., further postbaked treatment of the Pristine-0.25 h), (c) Pristine-20 h (i.e., synthesis of PUF in 20 h), and (d) Postbaked-20 h (i.e., further postbaked treatment of the Pristine-20h). By inspecting the representative intensities for the isocyanate peak from the MDI monomer (ca. 2270 cm^−1^) and the carbonyl peak from the PU backbone (ca. 1710 cm^−1^), we can estimate their relative conversion (rConv) [36]. For the samples prepared via the first step polymerization at 60 °C (i.e., curves a and c), we detected only a slight difference in the rConv values (i.e., 0.5 and 0.53, respectively), indicating the first step polymerization herein was insufficient to achieve high conversions. By conducting the second step further, the postbaked samples were acquired. The rConv values of (b) Postbaked-0.25h and (d) Postbaked-20h were dramatically increased to 0.69 and 0.95, respectively. It is thus plausible that the postbaked treatments can increase the reactions among the residual reactive functional groups and densify the packing of the polymer chains. Such post-mature processes for PU are important for our later practical evaluations toward the CMP process.

### 3.2. Thermal and Surface Properties of the PUF and MCC/NCC Composites

**Figure 4:**
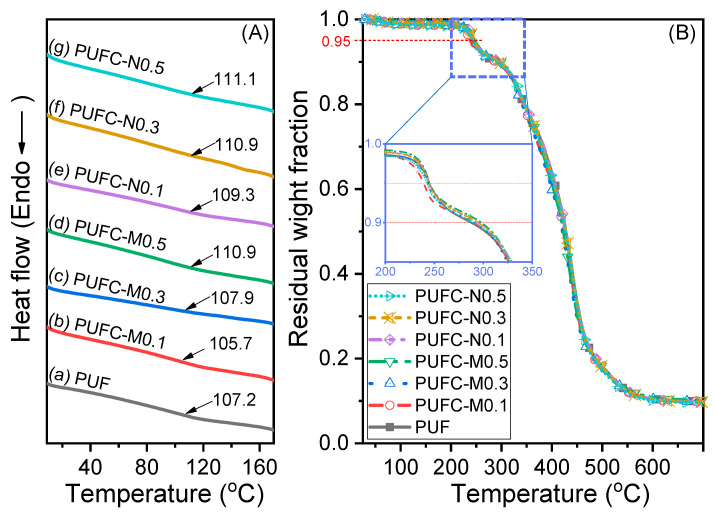
(**A**) DSC traces (2nd runs with 10–170 °C) and (**B**) TGA profiles (25–700 °C) of PUF and various PUFCs (ramp: 20 °C in N_2(g)_).

Then, PUF, MCC, and NCC composites were prepared through a two-stage mixing process (see Experimental section). PUF composites of the PUFC-M# and PUFC-N# series (# = 0.1, 0.3, and 0.5 (in phr)) were thus obtained. All the prepared samples displayed similar FTIR absorbance spectra (summarized in Figure S1 (see the Electronic Supporting Information (ESI)). We then used DSC to analyze the glass transition temperature (*T*_g,DSC_) of the PUFC-M and PUFC-N composites, as displayed in Figure 4A. The traces reveal that while the PUF is well mixed with the MCC/NCC, the *T*_g,DSC_ shows slight changes (i.e., *T*_g,DSC(PUF)_ = ca. 107 °C and *T*_g,DSC(PUFC)_ = up to ca. 111 °C). In some cases, the increases in *T*_g,DSC_ can be attributed to the interface effects between the MCC/NCC and the PU matrix, which may cause constraints of the polymer chains or stress, thereby reducing the chain mobility. The corresponding PUFCs were tested by thermogravimetric analysis (TGA). Adding additives during the PU polymerization (herein, MCC or NCC) might cause unexpected side reactions and decrease thermal stability. As shown in Figure 4B, however, TGA traces of all PUFC samples showed similar thermal stabilities, such as 5 wt% thermal degradation temperature (*T*_d5%_) and a char yield from room temperature to 700 °C. As revealed in the inserted figure, we can further observe that the addition of the MCC/NCC improved the thermal stability of *T*_d10%_ (i.e., *T*_d10%(PUF)_ = 277 °C and *T*_d10%(PUFC)_ = up to ca. 298 °C). In brief, Figure 4 provides an affirmative outcome to the thermal properties of the PUFCs with various MCC/NCC contents. The relevant results are summarized in Table 1. 

**Figure 5:**
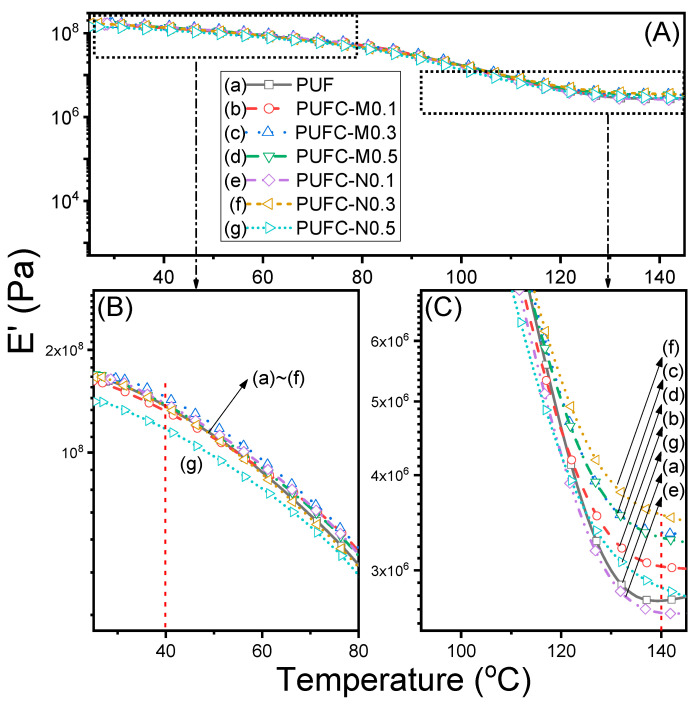
E′ profiles of PUF and different PUFC-M and PUFC-N samples: (**A**) 25–150 °C, (**B**) 25–80 °C, and (**C**) 90–150 °C (M: MCC; N: NCC; ramping 5 °C/min).

**Figure 6:**
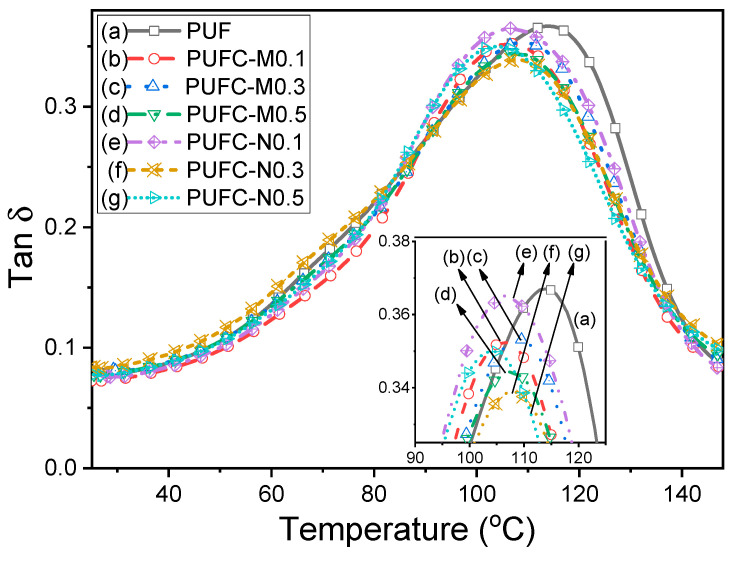
Tan δ curves of PUF and different PUFC-M and PUFC-N samples (M: MCC; N: NCC; ramping 5 °C/min from 25 to 150 °C).

**Figure 7:**
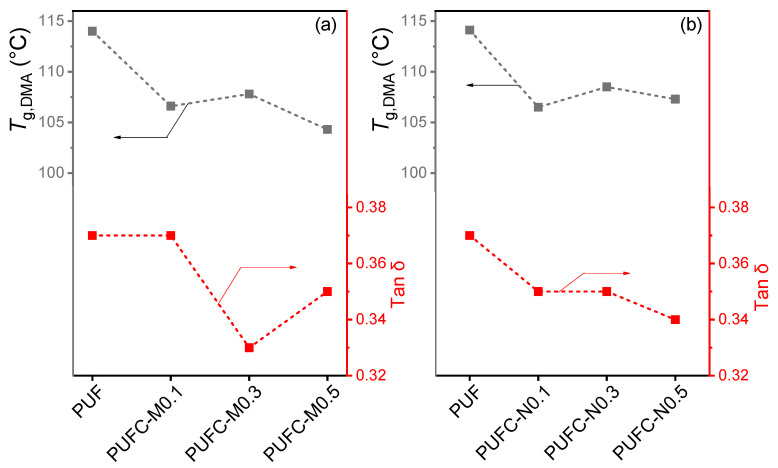
**Trends of E**’ and Tan δ vs. PUF and different (**a**) PUFC-M and (**b**) PUFC-N samples (M: MCC; N: NCC).

We then conducted DMA tests on the PUFC samples to realize the influences of adding the MCC/NCC, and the corresponding results are summarized in Table 1. As shown in Figure 5A, the E′ profiles of most PUFC samples are similar to the PUF. Except for PUFC-M0.1 and PUFC-N0.5, as shown in Figure 5B, the other PUFCs possess slightly higher E′ values than the PUF. We further compared the E′ values at a high temperature of 140 °C (i.e., after *T*_g,DMA_). As shown in Figure 5C, we observed that most of the PUFCs show significantly larger E’ than that of PUF, which might also be ascribed to the constraint effect from the MCC/NCC. During the CMP process, the wearing interfacial should be subjected to mechanical forces and chemical reactions, generating heat at the microscopic level. Therefore, such enhancement at a high temperature might be essential for practical CMP uses. Figure 6 presents the Tan δ curves for the PUF and PUFC samples, with the temperatures at the peak values of these curves representing the *T*_g,DMA_ (i.e., estimated from the x-axis). Similarly to the DSC results, the values of *T*_g,DMA_ in Figure 6 and Table 1 showed only slight differences. As shown in Figure 6 and Table 1, however, the maximum value of Tan δ_(PUFC-N0.3)_ (=0.33) (i.e., estimated from the y-axis) shows a significant decrease (ca. 11%) compared to that of Tan δ_(PUF)_ (=0.37). We recapitulate the trends among the *T*_g,DMA_, Tan δ, and MCC/NCC contents, as shown in Figure 7. It can be observed that the addition of the MCC/NCC resulted in a slight decrease in *T*_g,DMA_ (max. ca. 6%) but a reduction in Tan δ (max. ca. 11%), indicating an improvement in the material’s elasticity. This enhancement is attributed to the strong interface between the MCC/NCC and the PUF, which allows stress to be more effectively transferred within the composites. Rationally, the surfaces of the MCC/NCC containing many hydroxyl groups provide a physically compatible and chemically reactive interface that improves the overall physical properties of the polymer matrix.

**Figure 8:**
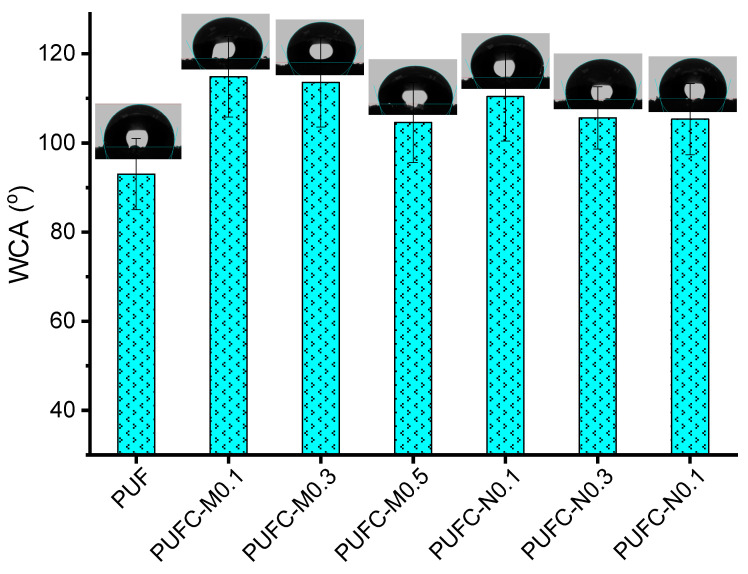
WCA (°) of PUF and various PUFCs (M: MCC; N: NCC; #: the added amounts of MCC/NCC (phr)).

Surface property changes are monitored by water contact angle (WCA (°)) measurements of the PUF and PUFCs, as shown in Figure 8. The results show a clear increase in WCA in the presence of MAA/NCC, with the angle rising from the initial hydrophobic value of around 92° for PUF to a significantly more hydrophobic range of approximately 105–115°. This elevation can be attributed to the synergistic micro/nano-binary structure (MNBS) combining the PUF and MCC/NCC [37,38,39,40]. By introducing MCC/NCC in good compatibility, a hierarchical architecture on the surface of the PUFCs can be easily attained. Having a large number of surficial hydroxyl groups of MCC/NCC with high crystallinity, as observed from the results mentioned above, the MCC/NCC can not only exhibit good compatibility within the PUF matrix but also serve as a stiff nano-fillers to enhance the thermal and mechanical and surface properties. In addition, smooth cross-sectional surfaces can be observed from the SEM images (displayed in Figure S2 (see the ESI)).

### 3.3. Mechanical and Abrasion Properties

**Figure 9:**
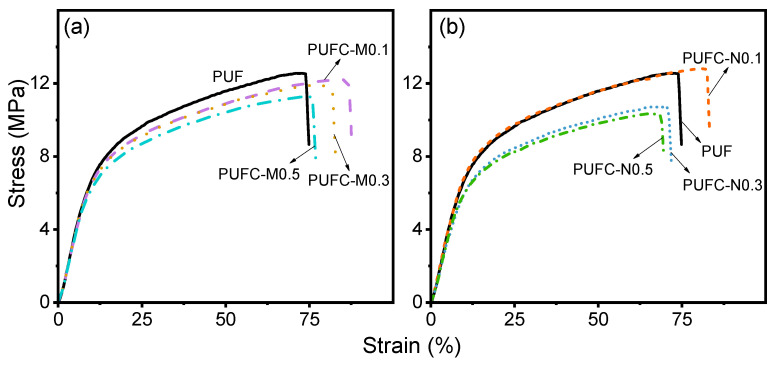
Tensile tests of PUF and different (**a**) PUFC-M and (**b**) PUFC-N composites (M: MCC; N: NCC).

With the well-dispersed MCC/NCC in the PUF matrix, tensile tests are conducted to reveal their stress–strain behaviors. With a certain amount of MCC (i.e., Figure 9a), the obtained mechanical properties of the PUFC-M exhibited a similar Young’s modulus and σ_m_ and an increased ε_m_ and *U*, compared to those of the PUF (σ_m(PUF)_ = 12.6 MPa, ε_m(PUF)_ = 71%, Young’s modulus = 31 MPa, and *U*_(PUF)_ = 19 kJ/m^3^). In the case of adding NCC (i.e., Figure 9b), we observed obvious improvements in σ_m_, ε_m_, and *U* in the PUFC-N0.1 sample and slight decreases in the corresponding properties in the others. In the affirmative PUFC samples, the improvement in mechanical properties is attributed to forming a good wetting interface between the cellulose fibers and the PU matrix. In the opposed PUFC samples, on the other hand, the slight abatements in mechanical properties might be due to certain agglomeration of celluloses, resulting in reduced load transfer from the matrix to the filler and causing the material to become uneven. Therefore, while preparing composites with MCC/NCC incorporation into polymers, it is crucial to ensure uniform dispersion of the fillers and an appropriate amount to maximize their reinforcing effects.

**Figure 10:**
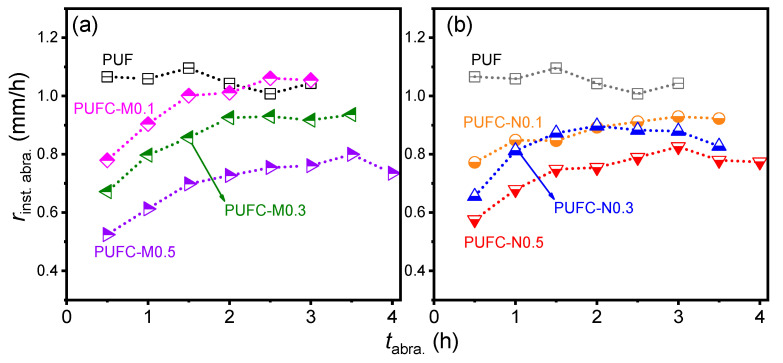
Plots of *r*_inst. abra_. vs pads with various *t*_abra._ for PUF and different (**a**) PUFC-M and (**b**) PUFC-N composites (M: MCC; N: NCC).

**Figure 11:**
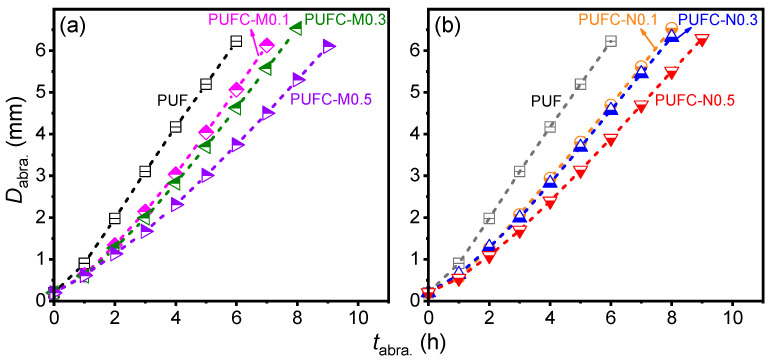
*D*_abra._ vs *t*_abra._ plots for PUF and different (**a**) PUFC-M and (**b**) PUFC-N composites (M: MCC; N: NCC).

As shown in Scheme 1, we performed general abrasion tests (conditions described in the Materials and Methods section) to evaluate and compare the PUF and PUFCs toward the practical CMP process. As shown in Figure 10, *r*_inst. abra._ values can be acquired. MCC/NCC might densify the PUF structures at the nanoscale, enhancing their abrasion resistance. Compared to the pristine PUF (*r*_inst. abra.(PUF)_ = 1.04 mm/h), almost all PUFC pads showed smaller *r*_inst. abra._ values (i.e., most cases have *r*_inst. abra._ < 1 mm/h). By further inspections, we can observe that the *r*_inst. abra._ deviation values in Figure 10a show a wider range (Δ*r*_inst. abra.(PUFC-M)_ = ca. 0.2) than those in Figure 10b (Δ*r*_inst. abra.(PUFC-M)_ = ca. 0.1), implying that more homogeneous PUFC-N samples were prepared. The values of and deviations in *r*_inst. abra._ are summarized in Table 2. Figure 11 shows the plots of *D*_abra._ vs *t*_abra._ for PUF and different PUFCs and we can acquire *r*_overall abra._ based on the slopes. As summarized in Table 2, all the PUFC specimens exhibit lower *r*_overall abra._ values than the pristine PUF (1.04 mm/h), indicating increasing abrasion resistance by the incorporation of MCC/NCC. The observed reduction in *r*_inst. abra._ values for the PUFC pads compared to pristine PUF can be attributed to the underlying physics of the material’s microstructure, particularly the role of MCC and NCC in reinforcing the PUF at the microscope. Including MCC/NCC leads to the densification of the foam, resulting in a more compact and uniform distribution of the polymer chains within the matrix [41]. From Table 2, the connections between the mechanical and abrasion tests showed an insignificant correlation. In this section, we demonstrate obvious enhancements in the mechanical properties and abrasion performance of the PUFCs, potentially providing a practical approach toward withstand longer periods of CMP use.

## 4. Conclusions

We inspected the MCC and NCC, which have average diameters of 140.9 ± 60.9 and 17.2 ± 5.7 nm, respectively. The highly crystallized MCC and NCC (ca. 89%) were well imposed into a PUF matrix, which has an optimized condition of postbaked 20 h through FT-IR monitoring on the variations of corresponding functional groups. After preparations of PUFCs, the *T*_g,DSC_ and *T*_d5%_ values only showed slight changes. However, *T*_d10%_ values showed significant differences between PUF (=ca. 277 °C) and PUFCs (=up to ca. 298 °C). In DMA analysis, E′ profiles of most PUFC samples are also similar to the PUF. Interestingly, most of the PUFCs manifested a larger E’ than that of PUF, which might retain their restrictiveness from MCC/NCC at a high-temperature region (e.g., 140 °C). The incorporation of MCC/NCC led to a slight decrease in *T*_g,DMA_ and a reduction in Tan δ (up to 11%), suggesting an enhancement in the material’s elasticity. WCA measurements increased from the original hydrophobic PUF (ca. 92°) to a more hydrophobic range (ca. 105–115°). This increase is plausibly due to the synergistic micro–nano binary structure of PUF combined with MCC/NCC. The tensile tests enhance mechanical properties due to forming a well-wetted interface between the cellulose fibers and the PU matrix. Using abrasion tests to evaluate and compare the PUF and PUFCs toward practical CMP process, almost all PUFC pads showed smaller *r*_inst. abra._ values. The deviation values of the PUFC-M showed a wider range than those of the PUFC-N, implying that more homogeneous PUFC-N samples were attained. By further comparing the *r*_overall abra._, the incorporation of MCC/NCC displayed increasing abrasion resistance. Eventually, we demonstrated significant improvements in the mechanical properties and abrasion resistance of the PUFCs, offering a practical solution for extending their durability in CMP applications. We anticipate that PUFC pads will potentially evolve to address more emerging process challenges and introduce new materials such as high-k dielectrics and 3D NAND technology.

## Figures and Tables

**Table 1:** Thermal properties of PUF and PUFCs.

Sample	*T*_g,DSC_ (°C)	*T*_d5%_^1^ (°C)	*T*_d10%_^1^ (°C)	Char Yield (%)	E’@40 °C (×10^−8^ Pa)	E’@140 °C (×10^−6^ Pa)	*T*_g,DMA_^2^ (°C)	Tan δ
PUF	105.8	244.3	277.5	9.9	1.35	2.72	114.1	0.37
PUFC-M0.1	105.7	239.2	290.3	9.8	1.31	3.02	106.5	0.35
PUFC-M0.3	107.9	244.4	290.1	9.8	1.45	3.38	108.5	0.35
PUFC-M0.5	110.9	245.4	297.9	10.1	1.37	3.32	107.3	0.34
PUFC-N0.1	109.3	245.3	294.2	9.7	1.39	2.64	106.6	0.37
PUFC-N0.3	110.9	243.8	294.4	9.7	1.35	3.56	107.8	0.33
PUFC-N0.5	111.1	243.4	290.4	9.5	1.16	2.83	104.3	0.35

^1^ *T*_d5%_ and *T*_d10%_ are the temperature at 5 wt% and 10 wt% degradations. ^2^ *T*_g,DMA_ is the temperature at the maximum value of the Tan δ trace.

**Table 2:** Summary of tensile and abrasion tests of PUF and PUFCs.

Sample	Max. Stress (σ_m_) ^1^ (MPa)	Max. Strain (ε_m_) ^1^ (%)	Young’s Modulus ^1^ (MPa)	Toughness (*U*) ^1^ (kJ/m^3^)	Avg. *r*_inst. abra._^2^ (mm/h)	*r*_overall abra._^3^ (mm/h)
PUF	12.6 ± 0.1	71.2 ± 4.4	31 ± 0.6	19 ± 1.0	1.05 ± 0.03	1.04
PUFC-M0.1	12.2 ± 0.4	84.2 ± 5.3	30 ± 1.1	21 ± 2.7	0.96 ± 0.11	0.98
PUFC-M0.3	11.9 ± 0.3	76.9 ± 4.5	31 ± 0.9	20 ± 2.1	0.86 ± 0.10	0.91
PUFC-M0.5	11.3 ± 0.1	73.5 ± 4.5	29 ± 0.8	17 ± 1.2	0.70 ± 0.09	0.76
PUFC-N0.1	12.8 ± 0.5	80.2 ± 4.9	31 ± 0.9	21 ± 3.1	0.87 ± 0.05	0.89
PUFC-N0.3	10.7 ± 0.3	68.5 ± 5.8	27 ± 0.5	15 ± 1.8	0.83 ± 0.08	0.85
PUFC-N0.5	10.3 ± 0.2	64.9 ± 3.4	28 ± 0.5	14 ± 0.9	0.74 ± 0.07	0.76

^1^ Estimated from the tensile tests. ^2^ Estimated from average values of the instantaneous abrasion rates in Figure 10. ^3^ Estimated from the tests of overall abrasion rates in Figure 11.

## Data Availability

Data is contained within the article and Supplementary Material.

## Supplementary Materials

The following supporting information can be downloaded at: https://www.mdpi.com/article/10.3390/polym16192738/s1, Figure S1: FTIR spectra (4000–600 cm^−1^) of (a–c) PUFC-Ms and (d–f) PUFC-Ns; Figure S2: SEM images (×40,000) from the cross-sections of (a) PUF, (b–d) PUFC-Ms, and (e–g) PUFC-Ns; Table S1: Summary of the prepared PUF and PUFC samples.
