# Supplementary material for: Preparations of Polyurethane Foam Composite (PUFC) Pads Containing Micro-/Nano-Crystalline Cellulose (MCC/NCC) toward the Chemical Mechanical Polishing Process
Source: Polymers (Basel). 2024 Sep 27;16(19):2738. doi: 10.3390/polym16192738 (PMC11479108; doi:10.3390/polym16192738)
Supplement: Supplementary file 1 [file polymers-16-02738-s001.zip › polymers-3205491-supplementary.pdf]

Electronic Supplement Information (ESI) for

Preparations of Polyurethane Foam Composite (PUFC) Pads Containing Micro-/Nano-crystalline Cellulose (MCC/NCC) Toward the Chemical Mechanical Polishing Process

Yi-Shen Huang [†1], Yu-Wen Huang [†2,3], Qiao-Wen Luo [1], Chao-Hsing Lin [3], Penjit Srinophakun [4], Supanicha Alapol [4], Kun-Yi Andrew Lin [5,*], and Chih-Feng Huang [1,2,*]

[1] Department of Chemical Engineering, i-Center for Advanced Science and Technology (iCAST), National Chung Hsing University, Taichung 40227, Taiwan; yishen617@gmail.com (Y.-S.H.); s26005916@gmail.com (Q.-W.L.)

[2] Semiconductor and Green Technology Program, Academy of Circular Economy, National Chung Hsing University, Nantou City, Nantou County 540216, Taiwan; oooo321280@gmail.com (Y.-W.H.)

[3] IV Technologies Co. Ltd., Taichung 40755, Taiwan; eddie.lin@ivt.com.tw (C.-H.L.)

[4] Department of Chemical Engineering, Faculty of Engineering, Kasetsart University, Bangkok 10900, Thailand; fengpjs@ku.ac.th (P.S.); supanicha.a@ku.th (S.A.)

[5] Department of Environmental Engineering, Innovation and Development Center of Sustainable Agriculture & Research Center of Sustainable Energy and Nanotechnology, iCAST, National Chung Hsing University, Taichung 40227, Taiwan

([†] Y.-S.H. and Y.-W.H. contributed equally to this work as first author.)

* Correspondence: linky@nchu.edu.tw (K.-Y.A.L.); HuangCF@dragon.nchu.edu.tw (C.-F.H.)

Captions:

Table S1. Summary of the prepared PUF and PUFC samples.

Figure S1. FTIR spectra (4000–600 cm^{-1}) of (a–c) PUFC-Ms and (d–f) PUFC-Ns.

Figure S2. SEM images (×40,000) from the cross-sections of (a) PUF, (b–d) PUFC-Ms, and (e–g) PUFC-Ns.

Table S1. Summary of the prepared PUF and PUFC samples.

Sample [1]	Polyol [2] (g)	Isocynate [3] (g)	MCC [4] (phr)	NCC [4] (phr)
PUF	20	10	–	–
PUFC-M0.1	20	10	0.1	–
PUFC-M0.3	20	10	0.3	–
PUFC-M0.5	20	10	0.5	–
PUFC-N0.1	20	10	–	0.1
PUFC-N0.3	20	10	–	0.3
PUFC-N0.5	20	10	–	0.5

[1] PUF: polyurethane foam; PUFC: PUF composite.
[2] Provided by iVT Ltd. Co. with the spec of C0-PO2024.
[3] Provided by iVT Ltd. Co. with the spec of C0-IS02024.
[4] MCC: microcrystalline cellulose; NCC: nanocrystalline cellulose.

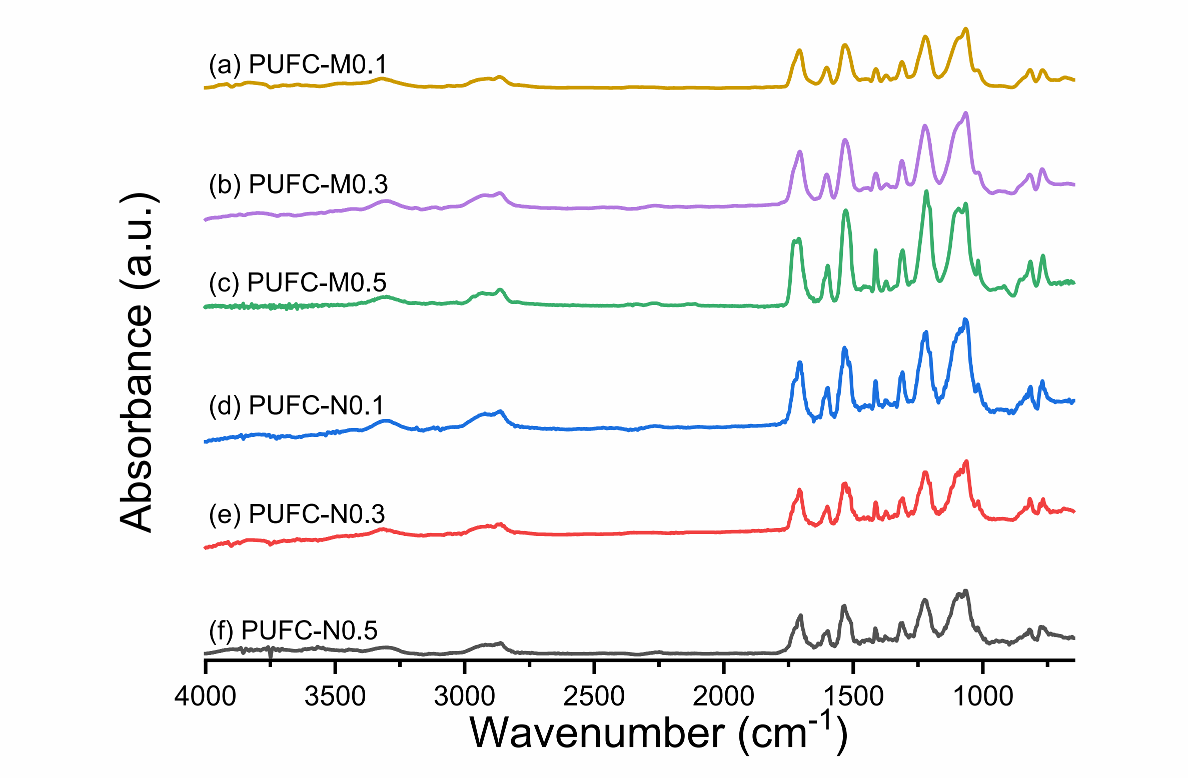

Figure S1. FTIR spectra (4000–600 cm^{-1}) of (a–c) PUFC-Ms and (d–f) PUFC-Ns.

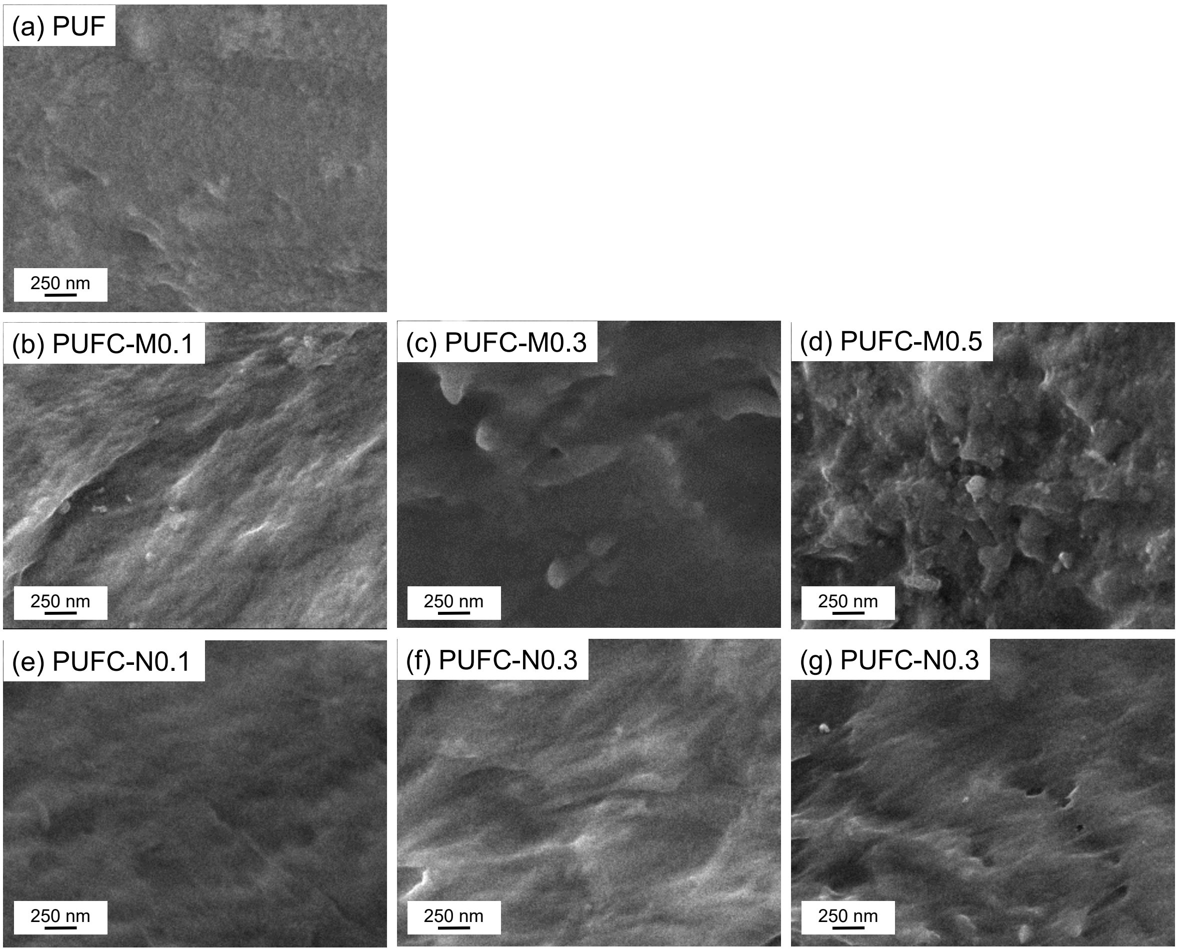

Figure S2. SEM images (×40,000) from the cross-sections of (a) PUF, (b–d) PUFC-Ms, and (e–g) PUFC-Ns.
